# Cross comparison of alternative diagnostic protocols including substitution to the clinical sample, RNA extraction method and nucleic acid amplification technology for COVID-19 diagnosis

**DOI:** 10.3389/fmolb.2024.1445142

**Published:** 2024-08-23

**Authors:** Ismael Segura-Ulate, Navilla Apú, Bernal Cortés, Jordi Querol-Audi, Yamitzel Zaldívar, Carlos Alexander Ortega, Fernando Flores-Mora, Andrés Gatica-Arias, Germán Madrigal-Redondo

**Affiliations:** ^1^ Instituto de Investigaciones Farmacéuticas (INIFAR), Facultad de Farmacia, Universidad de Costa Rica, San José, Costa Rica; ^2^ Agencia Costarricense de Investigaciones Biomédicas (ACIB) - Fundación INCIENSA (FUNIN), San José, Costa Rica; ^3^ Laboratorio de Microbiología Experimental y Aplicada (LAMEXA), Universidad de Panamá, Ciudad de Panamá, Panama; ^4^ Sistema Nacional de Investigación (SNI), SENACYT, Ciudad de Panamá, Panama; ^5^ Instituto Conmemorativo Gorgas de Estudio de la Salud, Ciudad de Panamá, Panama; ^6^ Sección de Virología, Facultad de Medicina, Universidad de El Salvador, San Salvador, El Salvador; ^7^ Escuela de Biología, Universidad de Costa Rica, San José, Costa Rica

**Keywords:** RT-LAMP, COVID-19, saliva sample, alternative protocol, SARS-CoV-2

## Abstract

**Background:**

the gold-standard diagnostic protocol (GSDP) for COVID-19 consists of a nasopharyngeal swab (NPS) sample processed through traditional RNA extraction (TRE) and amplified with retrotranscription quantitative polymerase chain reaction (RT-qPCR). Multiple alternatives were developed to decrease time/cost of GSDP, including alternative clinical samples, RNA extraction methods and nucleic acid amplification. Thus, we carried out a cross comparison of various alternatives methods against GSDP and each other.

**Methods:**

we tested alternative diagnostic methods using saliva, heat-induced RNA release (HIRR) and a colorimetric retrotranscription loop-mediated isothermal amplification (RT-LAMP) as substitutions to the GSDP.

**Results:**

RT-LAMP using NPS processed by TRE showed high sensitivity (96%) and specificity (97%), closely matching GSDP. When saliva was processed by TRE and amplified with both RT-LAMP and RT-qPCR, RT-LAMP yielded high diagnostic parameters (88%–96% sensitivity and 95%–100% specificity) compared to RT-qPCR. Nonetheless, when saliva processed by TRE and detected by RT-LAMP was compared against the GSDP, the resulting diagnostic values for sensitivity (78%) and specificity (87%) were somewhat high but still short of those of the GSDP. Finally, saliva processed with HIRR and detected via RT-LAMP was the simplest and fastest method, but its sensitivity against GSDP was too low (56%) for any clinical application. Also, in this last method, the acidity of a large percentage of saliva samples (9%–22%) affected the pH-sensitive colorimetric indicator used in the test, requiring the exclusion of these acidic samples or an extra step for pH correction.

**Discussion:**

our comparison shows that RT-LAMP technology has diagnostic performance on par with RT-qPCR; likewise, saliva offers the same diagnostic functionality as NPS when subjected to a TRE method. Nonetheless, use of direct saliva after a HIRR and detected with RT-LAMP does not produce an acceptable diagnostic performance.

## Introduction

The polymerase chain reaction (PCR) is the most employed nucleic acid amplification (NAA) technology for the targeted detection of nucleic acid sequences. However, since 1990 other NAA technologies have emerged, offering viable alternatives to PCR (Guatelli et al., 1990). Most of these alternative NAA methods are categorized as isothermal nucleic acid amplification technologies (INAATs), which offer the advantage of performing the entire assay at a constant temperature and eliminate the requirement of complex equipment like a thermal cycler. Additionally, INAAT may use in-tube colorimetric read-outs, providing instrument-free NAA options. This flexibility allowed the development of numerous INAAT-based point-of-care or field-deployable tests. INAATs also offer shorter turnaround times and lower costs than most PCR applications. All of which lead to the increased popularity for these alternative technologies in recent years (Fakruddin et al., 2013; Zhao et al., 2015).

Among the various INAATs, loop-mediated isothermal amplification (LAMP) stands out as the most widely utilized and extensively developed technology (Notomi, 2000). Since its inception LAMP has been used for a wide range of molecular detection applications, including in infectious diseases, genetic analysis, and food safety testing (Notomi, 2000; Mori et al., 2001; Mori and Notomi, 2009; Notomi et al., 2015; Li X. et al., 2022). Notably, the World Health Organization (WHO) developed a LAMP-based tuberculosis (TB) detection program known as TB-LAMP, created to provide affordable TB diagnosis for emerging economies (World Health Organization, 2016). During the pre-pandemic era, WHO’s TB-LAMP program represented the most extensive use of this NAA technology, and it provided the most comprehensive framework for comparing LAMP against PCR for a specific application. Head-to-head comparisons of LAMP and PCR tests for TB detection revealed that both methods exhibit similar diagnostic parameters when compared to the gold-standard diagnostic protocol (GSDP) for TB, namely, bacterial culture (Nagai et al., 2016; Babafemi et al., 2017; Phetsuksiri et al., 2020).

**FIGURE 1:**
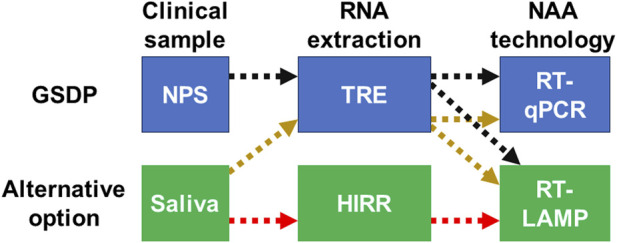
Schematic illustration of the accepted or traditional parts of the GSDP and the alternatives to each of these parts that are assessed in this study. Colored and dashed arrows show the combinations resulting in the different diagnostic protocols included in this comparison. GSDP, gold-standard diagnostic protocol; NPS, nasopharyngeal swab; TRE, traditional RNA extraction; HIRR, heat-induced RNA release; NAA, nucleic acid amplification; RT-LAMP, retrotranscription loop-mediated isothermal amplification; RT-qPCR, retrotranscription quantitative polymerase chain reaction.

The COVID-19 pandemic resulted in a global shortage of the GSDP for this novel infectious disease, which is composed of an RT-qPCR test from an NPS sample (Figure 1). As a result of the testing shortage, LAMP and other INAATs gained significant interest due to their flexibility and the potential for instrument-free testing. Throughout the pandemic, numerous commercial and in-house LAMP kits were developed as alternative options for COVID-19 detection, *in lieu* of the traditional retrotranscription quantitative PCR (RT-qPCR). Validation of these kits necessitated direct comparisons of retrotranscription LAMP (RT-LAMP) against the GSDP for COVID-19. These evaluations revealed that RT-LAMP exhibits sensitivity comparable, or very close, to that of RT-qPCR during the high viral load phase, which typically lasts up to 9 days after symptom onset. However, later in the infection, as well as in individuals with low viral loads, the sensitivity of RT-LAMP does not match that of the traditional RT-qPCR. Generally, comparative studies indicate that the raw sensitivity of RT-LAMP ranges from 80% to 90% when compared to the GSDP for COVID-19. Nevertheless, when individuals in the late stages of infection or those with a positive RT-qPCR test displaying high Ct values (e.g., >30–35^−ΔΔCT^) are excluded, the sensitivity of RT-LAMP increases to 90%–96%. Regarding specificity, RT-LAMP consistently performs at very high levels (i.e., 90%–100%) regardless of the infection stage or viral load (Baba et al., 2021; Inaba et al., 2021; Lu et al., 2022; Pu et al., 2022).

Following the identification of SARS-CoV-2 as a novel pathogen, NPS samples became the recommended clinical specimen for COVID-19 diagnosis. However, NPS sampling introduced workflow challenges that exacerbated testing deficits during the pandemic. Notably, NPS samples require traditional RNA extraction (TRE). Unfortunately, TRE methods are labor-intensive and require highly trained personnel or expensive automated RNA extraction platforms. Consequently, TRE from NPS added an additional bottleneck to the already overwhelmed COVID-19 testing workflow. In response, significant efforts were directed towards exploring the use of saliva as an alternative clinical sample. As a result, saliva is now recognized as an effective alternative to traditional NPS for COVID-19 diagnosis (Bastos et al., 2021; Lee et al., 2021; Okoturo and Amure, 2022). However, the change of the clinical sample by itself did not simplify the diagnostic test workflow. Thus, various studies assessed the use of saliva directly, after a quick heat-induced RNA release (HIRR) (LeGoff et al., 2021; Schneider et al., 2022). This quick method releases the viral RNA for NAA amplification by breaking virions’ structure. The HIRR method also inactivates virions, making the diagnostic test safer and simpler; but its biggest advantage is the elimination of the labor-intensive TRE.

Importantly, the combination of an alternative amplification technology (i.e., RT-LAMP) with an alternative clinical sample (i.e., saliva) and an alternative RNA extraction method (i.e., HIRR) introduces a complex array of possible protocols that require clinical validation for each substitution (Figure 1). For a more comprehensive review of all these alternatives in the detection of SARS-CoV-2, see the article by the Global LAMP (gLAMP) Consortium (Moore et al., 2021).

The primary objective of this paper is to determine the clinical diagnostic parameters resulting from replacing certain steps of the GSDP for COVID-19 with alternative methods, namely RT-LAMP, saliva samples, and quick HIRR (Figure 1). Specifically, this analysis focuses on the utilization of a colorimetric RT-LAMP test using saliva as a clinical sample, either after TRE or quick HIRR. To achieve this objective, we created a collaborative effort among research groups at Universidad de Costa Rica and Agencia Costarricense de Investigaciones Biomédicas (ACIB-FUNIN), utilizing the VALIDO study as the main platform for the assessment. Additionally, other Central American research centers, namely the Instituto Conmemorativo Gorgas de Estudios de la Salud in Panamá and the Universidad de Panamá, as well as the Universidad de El Salvador, participated in specific comparisons of this extensive set of tests.

## Materials and methods

### Populations and sample collection

In Costa Rica, NPS and saliva samples were collected as part of the VALIDO study, approved by the ACIB-FUNIN Institutional Ethics Review Board. Adults with a RT-qPCR COVID-19 test result within the past 5 days were enrolled in the study. Every participant signed an informed consent and provided both NPS and saliva samples. In total, 178 participants were enrolled between December 2020 and May 2021.

All participants of the VALIDO study were instructed to abstain from eating, drinking, or oral hygiene activities 30 min prior to saliva collection. Saliva was self-collected in a sterile 10 mL tube. NPS samples were collected by a qualified technician following standard procedure and preserved in Viral Transport Medium (VTM). All samples were kept in a cold box until their arrival at the laboratory during the same day. Samples were aliquoted into 1 mL vials and stored at −80°C until further processing. One aliquot from each sample was tested using validated procedures. Among the 178 participants, 99 were confirmed as positive by NPS by the sample collection time.

The instructions for saliva collection in the VALIDO study established that the sample should be, if possible, only liquid saliva without mucus or flehm mixed in. However, many of these samples did contain some amount of mucus or flehm. Thus, all procedures using these saliva samples began with a quick spin in a tabletop mini centrifuge to separate liquid saliva (lower) phase from any mucus or flehm contained in the sample. Only the liquid saliva (lower) phase of these samples was used for either TRE or HIRR.

In El Salvador, samples were collected from a group of 41 adults (21 years old or older) composed of students and personnel of the Universidad de El Salvador, who volunteered to participate in the study and provided an informed consent. Inclusion criteria included individuals with or without COVID-19 symptoms, as well as those with contact with a confirmed COVID-19 case, during the 14 days before the sample collection. Participants were instructed to abstain from eating, drinking anything other than water, oral hygiene and tobacco or vaporizers for 1 h prior to saliva collection. At least 2 mL of a sample composed of saliva and pharyngeal secretions was collected in a sterile 14 mL tube. Therefore, wherever a reference is made to saliva samples used at the El Salvador setting, it means that the sampe is composed of saliva plus pharyngeal secretion. All samples were kept in a cold box until their arrival at the laboratory during the same day. Samples were stored at −20°C until further processing.

In Panama, saliva samples were collected at the Instituto Conmemorativo Gorgas de Estudios de la Salud. Participants were instructed to abstain from eating or drinking 1 h prior to saliva collection. Samples were collected in a sterile 50 mL Falcon tube. This sample collection and all following procedures were approved by the Comité Nacional de Bioética de la Investigación.

### TRE for NPS and saliva samples

In Costa Rica, RNA extraction from NPS and saliva samples from the VALIDO study was performed by a semi-automatic procedure developed in-house using a Viaflow-96 liquid handler (INTEGRA Bioscience, Hudson, NH, United States) coupled with the MagMAX™ Viral/Pathogen Nucleic Acid Isolation Kit (Cat. A42352, Applied Biosystems, MA, United States).

In Panama, total RNA was extracted from 200 µL of saliva samples using the MagMAX Cell-free total RNA/DNA nucleic acid extraction kit (Cat. A36716, Applied Biosystems, MA, United States) in a KingFisher Flex System (Thermo Fisher Scientific, MA, United States).

In El Salvador, total RNA was manually extracted from 100 µL of saliva samples using the PureLink™ Viral RNA/DNA Mini Kit (Cat. 12280050, Thermo Fisher Scientific, MA, United States).

### RT-qPCR test from saliva using RNA extracted from saliva

In Costa Rica, RT-qPCR SARS-CoV-2 detection from both NPS and saliva samples from the VALIDO study were performed using TaqPath COVID-19 Fast PCR Combo Kit 2.0 (Cat. A51606, Thermo Fisher Scientific, MA, United States) and thermocycling was carried out in a QuantStudio 5 Real-Time PCR (Applied Biosystems, MA, United States). To assess laboratory competency for this in-house RT-qPCR test, 38 positive and 41 negative NPS samples were tested at our research laboratory as well as a clinically certified reference laboratory. The reference laboratory used Allplex™ 2019 nCoV RT-PCR kit (Cat. RP10243X, Seegene, Seoul, South Korea) following manufacturer instructions. This competency validation of the RT-qPCR at research laboratory against the clinically certified laboratory was blinded for the personnel of both settings, and it achieved a 100% agreement.

In Panama and El Salvador, RT-qPCR tests used the Charité Institute’s SARS-CoV-2–specific RT-qPCR protocol, including both the E and RdRp genes of SARS-CoV-2 (Corman et al., 2020). In both Panama and El Salvador, RT-qPCR tests were performed using the One-Step RT-PCR kit AgPath-ID (Cat. 4387424, Applied Biosystems, MA, United States). In Panama, thermocycling was carried out in an ABI 7500 Fast Dx Real-Time PCR (Applied Biosystems, MA, United States). In El Salvador, thermocycling was performed with a QuantStudio 5 Real-Time PCR (Applied Biosystems, MA, United States).

### RT-LAMP

RT-LAMP tests performed in all research centers used the SARS-CoV-2 Rapid Colorimetric LAMP Assay Kit (Cat. E2019S, New England Biolabs, MA, United States) following the manufacturer’s instructions, with a single modification: the guanidine HCL provided with the kit was not included in the reaction mix and the volume of this reagent was replaced with ultrapure water.

### Quick HIRR with saliva samples

HIRR was performed by incubating approximately 50 µL of liquid saliva at 95°C for 10 min in a screw cap sample tube. Saliva was allowed to cool down on ice before mixing it with the RT-LAMP mix or stored at −80°C until tested. Due to the acidity of some of the directly tested saliva samples, 10% of the direct saliva samples tested in Costa Rica and 28% of this type of samples tested in Panama caused the phenol red indicator in the RT-LAMP mix to turn either orange or yellow immediately after mixing these components. In Panama, these acidic saliva samples were not tested; however, in Costa Rica, the pH of these low-pH saliva samples was neutralized by mixing 0.1 µL of 0.1 M NaOH into the solution composed of RT-LAMP mix plus the directly tested saliva sample. In this pH-correction step, if the color of the solution remained yellow or orange after adding 0.1 µL of the NaOH solution, the same step was repeated as many times as necessary until the RT-LAMP mix returned to its base red color. In the case of these pH corrections, the final volume of the RT-LAMP reaction was kept at 25 µL by subtracting the added NaOH volume from the ultrapure water. Thus, ultrapure water was added to the RT-LAMP reaction solution as the final reagent before incubation.

### Statistical analysis

Sensitivity and specificity values of the alternative testing protocols were determined by comparing them against the GSDP or against each other using a two-sided Fisher’s exact test specifically designed for diagnostic tests comparison and included in GraphPad Prism 8. The software´s default 95% confidence interval (CI) was used. The mean and 95% CI range values for sensitivity and specificity are reported in Tables 1, 2.

**TABLE 1:** Diagnostic comparison of colorimetric RT-LAMP against RT-qPCR using the different clinical samples (saliva or NPS) and RNA extraction methods (TRE or HIRR) with samples from the VALIDO study.

RT-LAMP		Compared to RT-qPCR		Sample size (n)	Diagnostic values			
Sample	Extraction	Sample	Extraction		Sens	95% CI	Spec	95% CI
NPS	TRE	NPS	TRE	147	0.96	0.89–0.99	0.97	0.90–0.99
Saliva	TRE	NPS	TRE	76	0.78	0.63–0.89	0.87	0.73–0.94
Saliva	HIRR	NPS	TRE	54	0.56	0.34–0.75	1	0.90–1.00
Saliva	TRE	Saliva	TRE	164	0.96	0.89–0.99	0.95	0.89–0.98

NPS, nasopharyngeal swab; TRE, traditional RNA extraction; HIRR, heat-induced RNA release; RT-LAMP, retrotranscription loop-mediated isothermal amplification; RT-qPCR, retrotranscription quantitative polymerase chain reaction; CI, confidence interval; Sens., sensitivity; Spec., specificity.

**TABLE 2:** Diagnostic comparison of colorimetric RT-LAMP tests using direct saliva samples after quick HIRR (Panama) or traditionally extracted RNA from saliva samples (El Salvador) against RT-qPCR tests using saliva samples after TRE.

Research center and sample size	RT-LAMP (saliva and HIRR) vs. RT-qPCR (saliva and TRE)			
Panama (n = 56)	Sensitivity	95% CI	Specificity	95% CI
	0.65	0.43–0.82	0.94	0.82–0.99

	RT-LAMP vs. RT-qPCR (saliva with pharyngeal secretion and TRE for both)			
El Salvador (n = 41)	Sensitivity	95% CI	Specificity	95% CI
	0.88	0.71–0.96	1.00	0.78–1.00

NPS, nasopharyngeal swab; TRE, traditional RNA extraction; HIRR, heat-induced RNA release; RT-LAMP, retrotranscription loop-mediated isothermal amplification; RT-qPCR, retrotranscription quantitative polymerase chain reaction; CI, confidence interval.

## Results

Testing with the GSDP the 178 samples from volunteers in the VALIDO Study (Costa Rica) found that 100 of them were positive for COVID-19. Dual saliva and NPS samples from these 178 volunteers were used to conduct a series of comparative tests. The diagnostic parameters found for these comparisons are summarized in Table 1. Briefly, for NPS detected by RT-LAMP showed a sensitivity of 0.96 (95% CI: 0.89–0.99) and specificity of 0.97 (95% CI: 0.90–0.99) compared to GSDP. Likewise, when saliva processed by TRE was used with both NAA methods, RT-LAMP achieved very high diagnostic performance values, with a sensitivity of 0.96 (95% CI: 0.89–0.99) and specificity of 0.95 (95% CI: 0.89–0.98) compared to RT-qPCR. When saliva samples processed by TRE were detected by RT-LAMP, this alternative protocol produced a mid-range sensitivity of 0.76 (95% CI: 0.63–0.89) and specificity of 0.87 (95% CI: 0.73–0.94) compared to the GSDP. However, when saliva samples processed by HIRR were detected by RT-LAMP, this alternative protocol yielded a low sensitivity of 0.56 (95% CI: 0.34–0.75) and a perfect specificity (1.00, 95% CI: 0.90–1.00).

The samples from volunteers at the Panama research center were used to compare RT-LAMP using direct saliva samples after HIRR against RT-qPCR from saliva samples with TRE. The diagnostic parameters found for this comparison are summarized in Table 2 upper half. Briefly, this comparative testing of those 56 individuals showed that saliva samples processed by HIRR were detected by RT-LAMP also exhibited a low sensitivity of 0.65 (95% CI: 0.43–0.82) and a high specificity of 0.94 (95% CI: 0.82–0.99). Finally, at the El Salvador research center we carried out a comparison between RT-LAMP and RT-qPCR using saliva samples subjected to TRE for both tests. The diagnostic parameters found for this comparison are summarized in Table 2 lower half. Briefly, this comparative testing with 41 individuals showed a high sensitivity of 0.88 (95% CI: 0.71–0.96) and a perfect specificity of 1.00 (95% CI: 0.78–1.00) for RT-LAMP when compared to RT-qPCR.

## Discussion

Notably, using NPS processed by TRE yielded remarkably high sensitivity and specificity for RT-LAMP compared to the GSDP (Table 1). These results further support previous reports demonstrating that RT-LAMP offers diagnostic performance for COVID-19 that is equal or close to those of RT-qPCR (Baba et al., 2021; Inaba et al., 2021; Lu et al., 2022; Pu et al., 2022). Others have calculated that including RNA extraction, reagents, consumables and labor, a RT-qPCR test for COVID-19 is 75% more expensive than a RT-LAMP (Iqbal et al., 2022); however, this calculation leaves out the expenses associated with acquiring and running a real-time thermal cycler. Thus, the accumulated evidence demonstrates that RT-LAMP may be considered as an alternative to RT-qPCR, providing similar diagnostic sensitivity and accuracy while offering potential advantages in terms of costs, speed, and flexibility. These characteristics make RT-LAMP a particularly appealing option for clinical settings that lack the resources to use costly instruments such as thermal cyclers.

Comparing RT-LAMP using saliva processed by TRE to the GSDP resulted in sensitivity and specificity levels for the alternative test that, while high, did not reach a level of equivalence to GSDP (Table 1). However, it is important to note that the sensitivity and specificity levels found for this alternative protocol are higher than the values reported for some accepted isothermal diagnostic methods such as Abbott ID Now COVID-19 (Aupaix et al., 2021). Therefore, the diagnostic performance of this specific alternative protocol may still be regarded high enough for the development of low-cost diagnostic tests.

We also conducted comparisons of some of these alternative methods against one of the most widely accepted replacements for the GSDP. Specifically, we compared the performance of RT-qPCR tests using RNA traditionally extracted from saliva samples against RT-LAMP tests using the same clinical sample and extraction method. This specific comparison was independently performed in two different countries: Costa Rica and El Salvador. The diagnostic parameters detected in Costa Rica demonstrated closely matched diagnostic performance between these two protocols (Table 1). Results from El Salvador show a perfect specificity for RT-LAMP compared to RT-qPCR; nonetheless, in this independent comparison RT-LAMP yielded a sensitivity that, although high, does not match the detection capabilities of RT-qPCR (Table 2, lower half).

Although the combination of saliva, HIRR and RT-LAMP potentially produces a diagnostic protocol that is fast, simple, instrument-free and low-cost, our results show that the diagnostic performance of this alternative method is not acceptable for any clinical application due to low sensitivity (56%) compared to the GSDP (Table 1). These results are consistent with previous studies reporting sensitivity values for this approach as low as 34% when compared to the GSDP (LeGoff et al., 2021; Brown et al., 2022). Also, the diagnostic performance of this type of alternative protocol has shown significant variability in previous studies, as exemplified by the findings of two independent evaluations of the EasyCoV kit. The EasyCoV kit is a direct saliva RT-LAMP protocol like the one we assessed in this study. Interestingly, these independent assessments of the same direct saliva RT-LAMP kit report very different sensitivity values of 34% and 85.9%, respectively. These contradictory findings further emphasize the need for careful evaluation, validation and standardization of any potential substitutions in diagnostic protocols. Similarly, our comparison between RT-qPCR using RNA extracted from saliva, against RT-LAMP using saliva with a quick HIRR, also yielded a low sensitivity value (68%) (Table 2, upper half). Both of our results demonstrate that saliva processed by HIRR is not an ideal combination of sample and RNA extraction method.

Additionally, the use of a direct saliva sample after a quick HIRR in a pH-sensitive colorimetric RT-LAMP mix posed challenges due to the considerable wide pH range found in saliva samples. The tests conducted at the Costa Rica research center using this combination of clinical sample and simplified processing method revealed that roughly 9% of the saliva samples caused the pH-sensitive colorimetric reporter (phenol red) to turn from red to orange or yellow directly upon contact. Among Panamá saliva samples, a much higher percentage (22%) were acidic enough to change the RT-LAMP master mix color directly upon initial contact. While population differences, participant behavior, sample collection procedures, and environmental conditions may all contribute to the disparity in the percentage of highly acidic saliva samples between both settings, we have not identified a specific reason for it. This disparity also highlights the unexpected challenges of standardizing this particular alternative protocol. Regardless of the underlaying cause, these acidic saliva samples would require this simplified testing protocol to include an alternative diagnostic method or a pH correction procedure like the one developed for this study (see Materials and Methods). In one of our settings (Panamá), the low pH saliva samples found (16 out of the total 72 samples collected) were excluded from the comparison, leaving only 56 viable samples for the direct-saliva testing (Table 2). The highly acidic saliva samples found in the VALIDO study (5 out of a total of 54 samples) were kept as part of the assessment (Table 1) after applying our own pH correction method (see Materials and Methods). Other studies have developed similar pH correction methods to circumvent this specific issue (Uribe-Alvarez et al., 2021). We developed our pH correction method using human saliva for which the pH had been artificially changed and then spiked with viral RNA (not shown); however, the low sensitivity values of this alternative diagnostic protocol as a whole render our saliva pH correction method mute; thus, we decided not to pursue a clinical validation for the correction of the pH in these saliva samples. Furthermore, any pH correction approach introduces more steps and elements to the workflow and potentially increases the variability of the assay.

With regards to the controversial use of direct saliva after an HIRR, our results do not support this overly simplified alternative protocol, at least when coupled to RT-LAMP. However, we recognize that minor modifications of this alternative protocol have yielded better results than ours (see Table 3). A few studies have demonstrated that the inclusion of a saliva lysis buffer with a reductive agent, such as dithiothreitol (DTT) or tris(2-carboxyethyl) phosphine hydrochloride (TCEP), helps this type of direct saliva RT-LAMP assays to achieve mid-range (60%–77.2%) sensitivity values compared to the GSDP (Uribe-Alvarez et al.; Kobayashi et al., 2021) (see Table 3). It is hypothesized that a reductive saliva lysis buffer inactivates RNAses present in the sample, thus preserving the integrity of the RNA and improving detection. Similarly, Proteinase K was included in one of these buffers with the objective of degrading the virions’ capsid, but proteases also have the beneficial effect of inactivating RNAses, which lead to a high sensitivity value (94%) compared to the GSDP (see Table 3) (Vogels et al., 2021). A specific RNA stabilization agent (i.e., RNASecure) was part of one of these alternative protocols that reached a high sensitivity value (85%) compared to the GSDP (see Table 3) (Li Z. et al., 2022). Other studies mixed the saliva sample with commercial lysis buffers before the HIRR resulting in sensitivity values ranging from 40.9% to 98.7% compared to the GSDP (Brown et al., 2022; Dewhurst et al., 2022) (see Table 3). However, we cannot determine the mechanisms offered by these commercial buffers due to their unrevealed proprietary compositions.

**TABLE 3:** Reports found in scientific literature for the diagnostic sensitivity of COVID-19 tests composed of direct-saliva processed by HIRR and detected by RT-LAMP when compared against the GSDP.

Study	RNase inhibitor, reduction agent or protease	Other components of the saliva stabilization buffer	Sensitivity of alternative test compared to GSDP (%)
LeGoff et al. (2021)	None	None	34
Brown et al. (2022)	Not specified as buffer components	RapiLyze buffer (OptiGene, Horsham, United Kingdom)	40.9
Uribe-Alvarez et al. (2021)	TCEP	EDTA, NaOH, Tergitol NP-10 and SDS	60
L’Helgouach et al. (2020)	None	None	72.7
Kobayashi et al. (2021)	DTT	Tris-HCl and guanidine hydrochloride	77.2
Li et al. (2022b)	RNAsecure	Proteinase K, TE buffer (Tris plus EDTA) or PBS	85
Schneider et al., 2022	None	None	85.9
Vogels et al. (2021)	Proteinase K	None	94
Dewhurst et al. (2022)	Not specified as buffer components	AviSal sample collection buffer (Hayat Genetics, İstanbul, Türkiye)	98.7

## Conclusion

In general, our results support the use of RT-LAMP as a substitute NAA method in replacement of RT-qPCR for SARS-CoV-2 RNA detection. RT-LAMP may represent a viable and cost-effective solution for clinical settings lacking the expensive equipment and resources to carry out RT-qPCR. Similarly, our results also support the use of saliva as an alternative clinical sample in the diagnostic tests for COVID-19, as long as the saliva sample is subjected to a TRE method. Numerous reports show that this non-invasive clinical sample may provide some advantages over the traditional NPS; for example, patients prefer saliva over NPS, as they rate it as more comfortable and easier to collect, potentially increasing public acceptance towards testing (Plantamura et al., 2021). Likewise, self-collected saliva samples may decrease the overall cost of testing by reducing the number of samples collecting personnel, as well as personal protective equipment required. Savings of saliva collection are estimated to $636,105 per 100,000 people tested, compared to NPS (Bastos et al., 2021).

Our results do not support the direct use of saliva processed through a quick HIRR step in the diagnosis of COVID-19. This particular simplified alternative test could offer the advantages of a very fast and low-cost test that could potentially be carried out with very basic laboratory equipment; however, its diagnostics performance is too low to be considered as a viable option. Scientific reports of very similar diagnostic protocols show a wide variation in clinical sensitivity even when using the exact same kit (Table 3), reflecting the difficulties of standardizing the method. Furthermore, we found that the combination of a direct-saliva sample coupled with the pH-dependent readouts (e.g., phenol red) commonly found in colorimetric RT-LAMP tests implies technical challenges that require complicated solutions involving more steps and standardization, which will likely introduce more variability and cost, thus canceling out the advantages of this simplified alternative protocol. For these reasons, we consider that non-pH-sensitive NAA reporters such as hydroxy naphthol blue (HNB) or fluorescent intercalating dyes are better suited for the implementation of any alternative method using a sample with a wide pH range such as direct-saliva.

Finally, based on our results and those reported in the literature (Table 3), we conclude that the inclusion of a lysis or RNA stabilization buffer would improve the diagnostic performance of any test using direct-saliva after a quick HIRR as a clinical sample for COVID-19 diagnosis.

## Data Availability

The raw data supporting the conclusions of this article will be made available by the authors, without undue reservation.
